# Editorial: Global excellence in inflammatory eye diseases: Europe

**DOI:** 10.3389/fopht.2023.1354084

**Published:** 2024-01-05

**Authors:** Claudia Fabiani, Marc De Smet

**Affiliations:** ^1^ Ophthalmology Unit, Department of Medicine, Surgery and Neurosciences, University of Siena and Azienda Ospedaliero-Universitaria Senese [European Reference Network (ERN) for Rare Immunodeficiency, Autoinflammatory, and Autoimmune Diseases (RITA) Center], Policlinico “Le Scotte”, Siena, Italy; ^2^ MicroInvasive Ocular Surgery Clinic, Lausanne, Switzerland

**Keywords:** uveitis, JIA, macular edema (ME), CAR-T, cytomegalovirus (CMV)

Transcending geographical limitations, global collaboration is the very foundation of scientific progress, with collective efforts forging new pathways of knowledge. (1, 2) Exhibiting a steadfast commitment to collaborative pursuits, Frontiers in Ophthalmology has meticulously compiled a special edition Research Topic that highlights the latest advancements in Inflammatory Eye Diseases from all corners of the globe. A Research Topic featuring internationally acclaimed researchers, this showcase of academic prowess exemplifies the collaborative spirit that is integral to modern scientific exploration.

Within the extensive sphere of Inflammatory Eye Diseases, European researchers have contributed significantly. This Research Topic provides a unique view of the advancements in this field from a specific region, with a nuanced perspective. It emphasizes the close-knit nature of the scientific community, where scholars from various origins contribute. Pushing the boundaries of knowledge in unison, people converge to collectively address challenges.

In this handpicked Research Topic, the first article offers a detailed overview of biologic remedies for Uveitis triggered by Juvenile Idiopathic Arthritis (JIA-U) (Dini et al.). This all-encompassing review, published on August 15, 2022 in Frontiers in Ophthalmology, presents a map that steers through the complex treatment situation for JIA-U, which is notably challenging, especially in the pediatric population. The authors leave no stone unturned by deeply exploring the effects of cytokine blockers and cell-targeted therapies, providing subtle perspectives into how healthcare improvements are reshaping the future of JIA-U’s prognosis. As a comprehensive source for researchers and practitioners alike, this review is an essential tool, setting the path for continuing research and therapeutic interventions.

Moving to the second article, an original research piece published on June 2, 2023, the focus shifts to a meticulous examination of risk factors for the development of macular edema in children with uveitis (Friling et al.). This retrospective study, involving 150 pediatric patients, represents a significant contribution to our understanding of pediatric uveitis. The authors clearly identify key factors influencing the onset of macular edema, shedding light on the complexity of this condition. This research not only increases our understanding of pediatric uveitis but also underscores the critical importance of early detection and vigilant follow-up to improve patient outcomes. The implications of these findings extend beyond the confines of the article influencing practice in clinical settings and laying grounds towards better care for patients.

In the realm of case reports, the third article stands as a unique and compelling contribution (Saban et al.). Published on July 3, 2023, this case report details a successful treatment approach for cytomegalovirus retinitis. The administration of cytomegalovirus-specific cytotoxic T lymphocytes (CTL) represents a promising advancement in immunotherapy, suggesting potential alternatives for scientific exploration and therapeutic applications. This singular case not only showcases the potential of personalized treatments but also underscores the dynamic nature of treatment strategies, particularly in the context of hematopoietic cell transplantation. This report holds significance not only for its individual implications but also for its potential to pave the way for further explorations into personalized immunotherapeutic interventions.

Navigating the contemporary landscape, the fourth article addresses a concern that has emerged in the wake of global events – herpetic anterior uveitis following COVID-19 vaccinations (Ott et al.). (3) Published on September 22, 2023, this article presents a case series, providing a thorough exploration of the temporal relationship between vaccination and herpetic eye disease. The authors delve into the intricacies of this relationship, posing critical questions regarding potential risks that warrant further investigation. In the backdrop of the ongoing global vaccination efforts, this article assumes heightened relevance, urging the scientific community to explore deeper into the potential implications of vaccinations on ocular health.

Concluding this rich and diverse Research Topic is a mini-review that delves into the future potential of CAR-Treg cell therapies in treating ocular autoimmune conditions (Abraham et al.). (4) Published on April 18, 2023, this mini-review takes readers on a journey through the proliferating field of cell therapy for ocular autoimmune conditions. Focusing on Chimeric Antigen Receptor modified T cell (CAR-T) therapy, the authors articulate the potential application of this cutting-edge approach in treating ophthalmic conditions. The mini-review not only synthesizes existing knowledge but also serves as a launchpad for discussions surrounding future strategies in applying CAR-Treg therapy in the treatment of ophthalmic conditions.

In essence, this Research Topic on Inflammatory Eye Diseases serves as a comprehensive tapestry woven by the collaborative efforts of European researchers. Each article contributes a thread to this intricate weave, offering unique insights and perspectives that collectively advance our understanding of these diseases. As we navigate the complex terrain of eye diseases, this Research Topic exemplifies the power of global collaboration, pushing the boundaries of knowledge and paving the way for innovations that hold the promise of improved patient outcomes.

## Author contributions

CF: Writing – original draft, Writing – review & editing. MDS: Writing – original draft, Writing – review & editing.
